# Adaptive Momentum-Based Motion Detection Approach and Its Application on Handoff in Wireless Networks

**DOI:** 10.3390/s90705715

**Published:** 2009-07-17

**Authors:** Tein-Yaw Chung, Yung-Mu Chen, Chih-Hung Hsu

**Affiliations:** Department of Computer Science & Engineering, Yuan Ze University, No. 135 Yuan-Tung Rd., Chung-Li, Taoyuan, 32003, Taiwan E-Mails: armor@netlab.cse.yzu.edu.tw (Y.-M.C.); charles@netlab.cse.yzu.edu.tw (C.-H.H.)

**Keywords:** exponentially weighted moving average, handoff, MRSS, momentum, motion detection, ping-pong effect, received signal strength

## Abstract

Positioning and tracking technologies can detect the location and the movement of mobile nodes (MNs), such as cellular phone, vehicular and mobile sensor, to predict potential handoffs. However, most motion detection mechanisms require additional hardware (e.g., GPS and directed antenna), costs (e.g., power consumption and monetary cost) and supply systems (e.g., network fingerprint server). This paper proposes a Momentum of Received Signal Strength (MRSS) based motion detection method and its application on handoff. MRSS uses the exponentially weighted moving average filter with multiple moving average window size to analyze the received radio signal. With MRSS, an MN can predict its motion state and make a handoff trigger at the right time without any assistance from positioning systems. Moreover, a novel motion state dependent MRSS scheme called Dynamic MRSS (DMRSS) algorithm is proposed to adjust the motion detection sensitivity. In our simulation, the MRSS- and DMRSS-based handoff algorithms can reduce the number of unnecessary handoffs up to 44% and save battery power up to 75%.

## 1. Introduction

With the rapid development of wireless network technologies, the next generation wireless network is expected to integrate various wireless access technologies, such as Ultra-Wideband, WLAN, WiMAX, UMTS and 3GPP LTE to provide an all-IP communication environment and support various IP-based services, such as WWW, VoIP, IPTV, vehicle safety inspection [1, 2], intelligent transport systems [3], security monitor [4, 5] and homecare health, to mobile users anytime anywhere. In the environment, communication systems with various coverages could overlap one another. When a multi-mode mobile node (MN) is moving out of the coverage of its current serving access point (AP) or base station (BS), it needs to perform a vertical handoff or a horizontal handoff to switch its connections from the current serving AP to another AP to avoid connection interruption. Therefore, the performance of handoff mechanism significantly affects the quality of communication services.

The handoff procedure can be divided into three stages [6]: handoff initiation, handoff decision and handoff execution. In the first stage, an MN turns on all its wireless interfaces to discover which wireless networks are available. Then, the MN selects the best network. Finally, the MN executes a handoff process to switch its connection from the current access network to the one newly selected while minimizing handoff delay. In the past, several network discovery schemes [7–27] have been proposed to reduce the handoff delay and the packet loss rate. These network discovery schemes can be classified into received signal strength (RSS) threshold-based methods [7–19] and motion-aware methods [20–27].

The RSS threshold-based schemes make an MN turns on all interfaces for network discovery only when the RSS of the current active interface is below a pre-defined threshold. However, power consumption and handoff dropping rate are in a tradeoff. For instance, if the RSS threshold is high, an MN will turn on its interfaces early for network discovery, which makes handoff smooth but increases the power consumption. On the contrary, if the RSS threshold is set to a low value, the MN turns on its interfaces late. Although the MN can consume less power, it may not have sufficient time to perform network discovery and handoff execution, which increases the handoff dropping rate.

The motion-aware network discovery method improves the performance of handoff by exploiting location information offered by Global Position System (GPS) [20], Inertial Navigation System (INS) [21] or location systems to predict the position and motion state of an MN. Unfortunately, the GPS system may not work well in an indoor environment and metropolitan area. Although indoor positioning systems [22–26, 28] can use an RSS map to help positioning an MN and detect the MN’s motion, it needs extraneous hardware and has high implementation cost.

This work proposes a motion detection scheme called Momentum of Received Signal Strength (MRSS) to detect an MN’s motion without the assistance of any positioning systems. With the MRSS method, an MN can turn on its interfaces to discover candidate wireless access networks only when it is leaving the service area of the current associated AP and terminate unnecessary handoffs when it is stationary, leading to significant reduction in power consumption and improvement in handoff success rate.

Although MRSS is sensitive to the MN’s motion, it is static and fails to quickly detect the changes in the MN’s motion. Thus, a novel motion state dependent MRSS scheme called Dynamic MRSS (DMRSS) algorithm is proposed to address this issue. Extensive simulation experiments were conducted to study the performance of our presented algorithms. The simulation results show that MRSS and DMRSS can substantially improve the motion detection delay and be utilized by a handoff algorithm to reduce unnecessary handoff and save battery power.

The rest of this paper is organized as follows. Section 2 overviews related network discovery schemes and motion detection technologies. Section 3 details the proposed Momentum of Received Signal Strength (MRSS) based motion detection method and the proposed MRSS-based handoff algorithm. Section 4 analyses the property of MRSS on various design options and evaluates the performance of MRSS-based handoff algorithm. Finally, Section 5 draws conclusions and discuss future works.

## 2. Related Works

Previously many network discovery approaches have been proposed. These network discovery approaches can be classified into RSS threshold-based methods and motion-aware methods. The RSS threshold-based methods include reactive methods and proactive methods. The proactive network discovery [6–14] uses a decision function based on a handoff mechanism. In the proactive scheme, an MN always turns on all of its wireless interfaces to monitor and scan the available access networks. If the MN finds out a better network, i.e., the RSS from the new AP is higher than that of the current serving AP, the MN triggers handoff process to switch its connection to the new AP. However, this method may cause ping-pong effect and reduce the performance of communication service. In [12], the authors use a dwell timer and an effective throughput ratio to optimize the RSS-based handoff algorithm while reducing the ping-pong effect. Chang *et al.* [14] propose an RSS prediction method and an adaptive cost-based competitive on-line (COL) method to trigger handoff and select a suitable wireless network. The predicted RSS is equal to the current RSS plus a difference value. The range of the predicted RSS is between the exponentially averaged RSS and the averaged RSS of the MN. If the predicted RSS is below a predefined threshold, the handoff will be triggered. However, these handoff algorithms do not consider power consumption.

The reactive approaches [15–18] use predefined thresholds to trigger an MN’s network discovery so that the MN can preserve battery power. In [18], the authors discuss network discovery by using Link Going Down (LDG) trigger in the Media Independent Handover (MIH) framework [19]. LDG is an event message that is triggered by an MIH Information Service (IS) according to the threshold used. LDG can be invoked by various approaches to prevent ping-pong effect. Zahran *et al.* [17] proposes an Application Signal Strength Threshold (ASST) mechanism, which defines different thresholds for applications. When the RSS slope estimation is smaller than ASST, network discovery is triggered. However, when many applications are active on an MN simultaneously, how to define thresholds becomes a problem.

The motion-aware methods use the MN’s location and motion information to trigger handoff. There are several location, tracking and motion detection technologies [20, 22–31], such as GPS, Assisted-GPS (A-GPS), time of arrival (TOA), time difference of arrival (TDOA), angle of arrival (AOA), OTDOA, cellular ID, smart antenna and pattern matching (network fingerprint [32]), have been proposed. However, these motion detection mechanisms require additional hardware and cost.

The GPS and A-GPS method use the GPS information to track an MN’s location and movement. An MN can receive GPS information from at least four satellites and the assistance data from network to accurately predict its location. However, the MN needs to equip a GPS receiver, which implies more monetary cost and more power consumption. Moreover, the GPS signal may not be received accurately in an indoor environment.

The TOA method measures the travel time of a radio signal from an MN to three different APs. Each travel time is multiplied by the light speed to give the distance between the MN and each AP. The TOA method can estimate the MN’s position and motion based on the intersection of these distance information. The TDOA method is an enhancement of TOA, which computes the MN’s position based on trilateration. The TDOA method uses the time difference measurement in the location prediction. However, the TDOA method requires a synchronized clock for all communication systems to measure the time difference, which makes the TDOA method an expansive solution. On the other hand, the AOA method needs to use an antenna array or a smart antenna to estimate the angle of received signal, which is not easy to implement in many wireless systems. In addition, all of the above methods require the cooperation between clients (MNs) and networks (APs) to monitor radio signal, measure location and predict motion.

In the cell-ID method, the sector of AP/BS will broadcast its sector information, thus an MN can use these information to identify its location. However, the accuracy of motion detection is limited by the size of the cell sector. The pattern matching method [32] uses a location server which contains a signal characteristics database to analyze the location of an MN. When an MN moves in a network, the MN measures the radio pattern (e.g., RSS, signal-to-noise ratio) from the current serving AP and sends the measured pattern to the location server. The location server will compare the radio pattern with signal characteristics database and identify the location of the MN. Then, the communication system can use this location information to predict the motion of the MN and improve the handoff process. However, the signal characteristics database must be constructed in advance, and hence the pattern matching method may not be usable in a highly variable environment.

The authors in [27] presented a motion detection algorithm based on Moving Average Convergence / Divergence (MACD) and also introduced an MACD-based handoff algorithm. The MACD technology is a trend-following indicator that is widely used in stock price prediction. The MACD-based handoff algorithm enables the MN’s interfaces when the MN is moving away from its current serving AP. The authors claim that the MACD-based motion detection approach can reduce 30 percent of battery power consumption than that of the always-on approach. However, because of the overlapped sample problem, the MACD-based motion detection algorithm is insensitive, especially when the MN is far away from the BS or moves at a slow speed.

## 3. Momentum-Based Motion Detection

The simplest method for detecting the user motion state is to use the received signal strength (RSS) analysis. RSS is a kind of time-series data. Since the RSS of an MN is related to the distance between an MN and its associated AP, the RSS value *P_r_* at the time *t* is given by

(1) $$P_{r}\;[t]=P_{\mathrm{tx}}-10\rho \;\text{log}\;[d]+X_{\mathrm{dB}}$$

where *d* is the distance between the MN and the associated AP, *P_tx_* is the transmitted signal power, *ρ* is the path loss exponent, and *X_dB_* is a Gaussian random variable with zero mean and standard deviation *σ_dB_* representing the shadow fading. According to Equation 1, the difference between two continuously measured RSS at distances *d*_1_ and *d*_2_ can be expressed as

(2) $$\Delta P_{r}\;[t]=P_{r}\;[t]-P_{r}\;[t-1]=-10\rho \;\text{log}\;\left[\frac{d_{2}}{d_{1}}\right]$$

Given the measured RSS interval and the direction and velocity of a user movement, the user motion can be identified based on Equation 2 as

(3) $$\text{user motion}\;=\;\{\begin{array}{ll} \text{stationary state}, & \text{if}\;\Delta P_{r}\;[t]=0\\ \text{approaching state}, & \text{if}\;\Delta P_{r}\;[t]>0\\ \text{leaving state}, & \text{if}\;\Delta P_{r}\;[t]<0 \end{array}$$

However, the measured RSS fluctuates constantly due to the fading effect. In the real world, the surrounding environment is always changing and hence RSS also changes continually. Even if the MN is stationary, the measured Δ*P_r_* [*t*] may not be zero. Therefore, an MN cannot easily detect user motion based only on Δ*P_r_* [*t*] without filtering fading noise.

### 3.1. MRSS Definition

MRSS is set as the difference between current smoothed RSS sample and early smoothed RSS sample. The smoothed RSS can eliminate fading noises. The computation process of MRSS can be divided into three steps. First, an Averaged RSS (ARSS) [33] is computed, which is the average of *T* samples in the *i^th^* interval of time:

(4) $${\mathrm{ARSS}}_{i}=\frac{\sum_{j=1}^{T}{\mathrm{RSS}}_{i\times T+j}}{T}$$

where *i >* 0, *T >* 0.

In the second step, an exponentially weighted moving average (EWMA) filter [33], a low pass filter, is applied to the signal analysis process to smooth the ARSS. The EWMA filter is defined as

(5) $$\mathrm{EW}\;MA_{i}=(1-\alpha )\times \mathrm{EW}\;MA_{i-1}+\alpha \times \;{\mathrm{ARS S}}_{i}$$

where *i* ≥ 1, 0 < *α* < 1. *EW MA_i_* is the current estimated EWMA value, *EW MA*_*i*−1_ is the prior estimated EWMA value, *ARSS_i_* is the current measured ARSS value, and *α* is a smooth factor which can filter the noise of ARSS and keep the EWMA stable.

The EWMA curve becomes more stable when the EWMA uses a smaller smooth factor (*α*), which is called the Long-term EWMA (L-EWMA) and can predict the trend of an MN’s motion. On the other hand, when EWMA uses a larger smooth factor (*α*), which is called the Short-term EWMA (S-EWMA), the S-EWMA curve changes quickly and can track the MN’s motion swiftly. The smaller *α* make the *EW MA_i−_*_1_ (old data) relatively more important than *ARSS_i_*, and the larger *α* discounts older observations quickly.

However, selecting a suitable *α* for an EWMA filter is difficult. Based on Equation 5, *EW MA_i_* can be easily expanded to

$$\begin{array}{ccc} \mathrm{EW}\;MA_{i} & = & {\left(1-\alpha \right)}^{0}\times (\alpha )\times {\mathrm{ARSS}}_{i}\\ & + & {\left(1-\alpha \right)}^{1}\times (\alpha )\times {\mathrm{ARSS}}_{i-1}\\ & & \vdots \\ & + & {\left(1-\alpha \right)}^{i-1}\times (\alpha )\times {\mathrm{ARSS}}_{1}\\ & + & {\left(1-\alpha \right)}^{i}\times {\mathrm{ARSS}}_{0} \end{array}$$

which can be written as follows:

(6) $$\mathrm{EW}\;MA_{i}=\left[\sum_{x=0}^{(i-s)-1}{\left(1-\alpha \right)}^{x}\times (\alpha )\times {\mathrm{ARSS}}_{i-x}\right]+{\left(1-\alpha \right)}^{\left(i-s\right)}\times \mathrm{EW}\;MA_{s}$$

where *i* > *s* ≥ 0, *i* − *s* > *x* ≥ 0.

In Equation 6, we assume *k* = *i − s*, *k* > *x* ≥ 0, *γ* = (1 *− α*)*^k^* and *σ_x_* = (1 *− α*)*^x^* *×* (*α*). Thus, Equation 6 can be written as follows:

(7) $$\mathrm{EW}\;MA_{i}=\left[\sum_{x=0}^{k-1}\sigma_{x}\times {\mathrm{ARSS}}_{i-x}\right]+\gamma \times \mathrm{EW}\;MA_{s}$$

where *i* > *s* ≥ 0.

Clearly, *k* is the sampling size (also called moving average window size) of an EWMA filter, *γ* is the impact factor of *EW MA_s_* on *EW MA_i_*. The EWMA filter assumes that when *γ* is smaller than 0.005, the impact of *EW MA_s_* on *EW MA_i_* can be ignored. Thus, the moving average window size can decide a suitable *α* in EWMA filters, which is computed by

(8) $${\left(1-\alpha \right)}^{k}<0.005$$

where *k >* 1. According the scaling function in multi-resolution analysis [34], the sampling size for a signal analysis is scaled by two, thus we can assume *k* = 2*,* 4*,* 8*,* 16*,* 32*,*⋯. Table 1 shows the suitable *α* value on various moving average window sizes.

**Figure 1.**
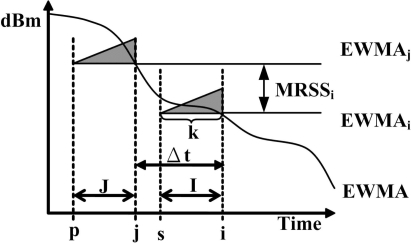
The MRSS calculation.

Finally, MRSS is defined as the difference between *EW MA_i_* and *EW MA_j_* as follows:

(9) $${\mathrm{MRSS}}_{i}=\mathrm{EW}\;MA_{i}-\mathrm{EW}\;MA_{j}$$

where *i* > *j* ≥ 0. Figure 1 shows the calculation of MRSS, where Δ*t* is the time difference between *EW MA_i_* and *EW MA_j_*.

### 3.2. Motion Detection Methodology

Based on the MRSS method, we can detect an MN’s behavior by two thresholds, the positive threshold (*T H_P_*) and the negative threshold (*T H_N_*). The state of an MN’s motion is then defined by the following rules:

- Approaching state: when MRSS is positive and rising above (*T H_P_*), the MN is considered to be approaching the BS.
- Stationary state: when MRSS is close to zero and stays between (*T H_N_*) and (*T H_P_*), the MN is considered to be staying at the same place.
- Leaving state: when MRSS is negative and declines below (*T H_N_*), the MN is considered to be leaving the BS.

**Figure 2.**
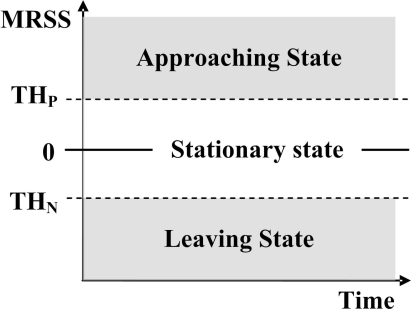
The detected user motion state of the MRSS method.

Figure 2 shows the detected user motion state obtained by the proposed MRSS scheme. Thus, by using the MRSS-based motion detection method, a motion detection based handoff algorithm can trigger a network discovery process at correct timing.

### 3.3. Dynamic MRSS

In the MRSS method, the smooth factor (*α*) is usually fixed and never changed. However, when the smooth factor is set according to an MN’s state of motion, the sensitivity of MRSS can be improved. Sensitive detection can determine the correct instant to execute or terminate a network discovery process, and prevent unnecessary power consumption. We proposed a Dynamic MRSS (DMRSS) scheme to improve the sensitivity of MRSS based on the following observations:

- Before an MN starts moving, EWMA only has a small fluctuation, hence using S-EWMA to detect the change of an MN’s motion state is easier than L-EWMA.
- When an MN is in the leaving/approaching state, EWMA has large fluctuations, thus using LEWMA to detect the change of an MN’s state is more accurate than S-EWMA.

DMRSS uses two different pairs of smooth factors (*α*) for various motion states of an MN. In DMRSS, when an MN is in the moving state (approach or leaving), EWMA uses a smaller smooth factor to make the EWMA curve smooth so that we will not ignore the MN’s state change. In the stationary state, EWMA uses a larger smooth factor to make the EWMA curve sensitive to RSS changes. Thus, when a MN starts moving, MRSS will quickly increase and make motion detection easy.

### 3.4. MRSS-based Handoff Algorithm

Based on the user motion state detection results, an MN can decide to activate or terminate its interfaces to save power and reduce handoff dropping rate in a network discovery process. In the MRSS-based handoff algorithm and the DMRSS-based handoff algorithm, a pre-defined handoff threshold (*T H_HO_*), a network discovery threshold (*T H_ND_*) and three network discovery modes are defined.

In the general threshold based handoff algorithm, when the EWMA of the current servicing base station is lower than *T H_HO_*, the MN must execute the handoff procedure immediately to avoid disconnection. When EWMA is lower than *T H_ND_*, the MN should perform network discovery. *T H_ND_* must be higher since an MN must turn on all of its interfaces in time to perform network discovery procedures such as base station searching, association, Authorization, Authentication and Accounting (AAA), IP address configuration, and other high layer signaling functions, before switching to a new network. However, using a high EWMA threshold certainly increases power consumption. Therefore, the following three network discovery modes are defined in the MRSS/DMRSS-based handoff algorithm to reduce power consumption.

- **NON_ND mode**: this mode is used when an MN is approaching an AP or BS. Therefore, network discovery is not needed.
- **ND mode**: this mode is used when an MN is leaving the associated AP or BS. Therefore, timely activation of interfaces is critical for detecting all available wireless networks.
- **SEMI_ND mode**: this mode is applied when an MN is stationary. If *EW MA < T H_HO_*, the MN must activate all of its interfaces to perform network discovery and handoff procedure. Otherwise, the MN terminates the network discovery process.

**Figure 3.**
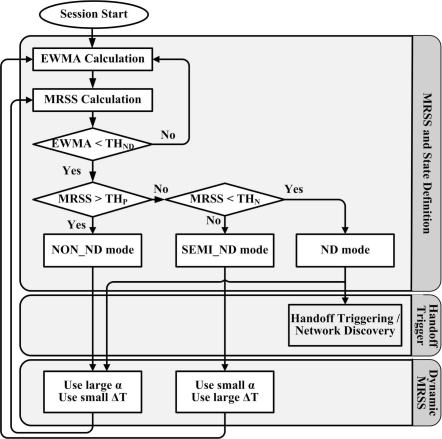
The flow chart of DMRSS-based handoff algorithm.

Figure 3 shows the flow chart of the DMRSS-based handoff algorithm. When an MN connects to an AP, the EWMA and MRSS are measured and the user motion is continuously determined. When the EWMA is smaller than *T H_ND_*, the MN checks its motion state. If the MN is in an approaching state, the network discovery mode is set to the NON_ND mode. If the MN is in a leaving state, the network discovery modes is set to an ND mode. If the MN is in a stationary mode, the network discovery modes is set to the SEMI_ND mode. During the network discovery process, a dwell-time mechanism [9] is utilized to avoid the ping-pong effect. If the MN is in the ND mode, continuously leaves the current serving AP and approaches the new AP until the dwell timer expires, the MN can switch its connections to the new AP.

## 4. Performance Evaluation

In this section, extensive computer simulations were conducted to study the performance of MRSS and dynamic MRSS (DMRSS). At first, the basic properties of MRSS were analyzed. Afterwards, the impact of approaching and leaving movements were studied. In the third simulation, the feasibility of the MRSS in a WLAN and a Mobile WiMAX [35] were examined. Then, the effect of the ping-pong effect were investigated using various mobility models. Finally, the performances of the MRSS-based handoff algorithm and DMRSS-based handoff algorithm were investigated.

**Figure 4.**
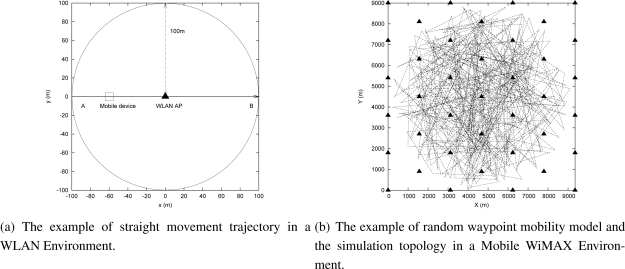
The mobility model and topology in simulations.

In all simulations, a log-normal shadowing model in NS2 simulator [36] and the BonnMotion node-movement generation tool [37] were used. A simple straight movement trajectory and the random waypoint mobility model were adopted to simulate a user movement trajectory. Figure 4(a) shows the example of the straight movement trajectory and Figure 4(b) shows the example of the random waypoint mobility model. In order to simulate various wireless environments, a WLAN and a Mobile WiMAX environment were chosen. Table 2 shows the default parameters of the log-normal shadowing model for radio propagation in all simulations.

### 4.1. Basic Property Analysis

In this analysis, the properties of the MRSS mechanism were evaluated by various smooth factors (*α*), moving average window sizes (*k*), shadowing deviations and velocities in a WLAN and a Mobile WiMAX environment. The simulation uses a simple straight movement trajectory as shown in Figure 4(a). The MN moves from location A to location B at a velocity of 1 m/s in a WLAN environment.

**Figure 5.**
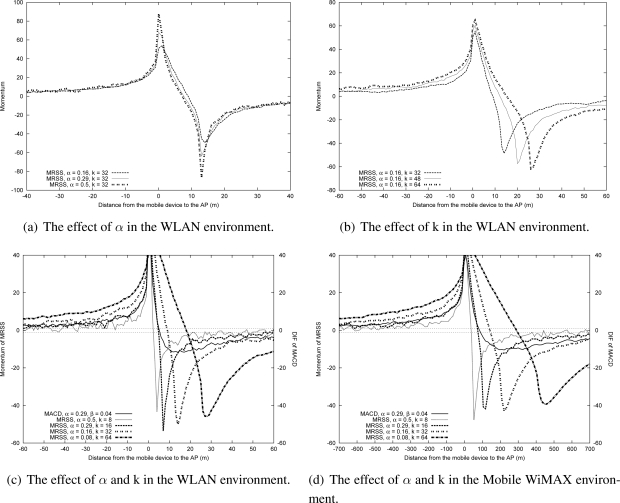
The performance evalution of *α* and *k*.

Figure 5(a) shows the effect of using various *α* with a fixed *k* on the momentum of the MRSS-based motion detection algorithm, where *k* represents the size of the moving average window. Figure 5(a) reveals that *α* barely affects the momentum value as a user approaches. Nevertheless, as a larger *α* is applied, the momentum of MRSS will reduce rapidly when the MN moves away from the AP. Thus, the MN can rapidly detect the user’s leaving state.

Figure 5(b) presents the effect of using various *k* with a fixed *α* on momentum. The value of *k* is related to the sample distance between two EWMA filters in MRSS. A larger *k* increases the motion detection delay in MRSS. When *α* = 0.16, the minimum value of *k* is 32. In this analysis, various *k* (*k* ≥ 32) were configured in the simulation. The simulation results confirm that using a suitable *k* can detect user motion more quickly and accurately. Besides, because of the effects of radio propagation, a longer distance between the MN and the AP also corresponds to a smaller momentum change rate in MRSS.

The results in Figure 5(a) and Figure 5(b) indicate that using a suitable *α* and *k* enables rapid and accurate identification of user motion state. Therefore, four (*α, k*) pairs, (*α* = 0.08*, k* = 64), (*α* = 0.16*, k* = 32), (*α* = 0.29*, k* = 16) and (*α* = 0.5*, k* = 8), are selected to evaluate the MRSS characteristics. In order to compare MRSS with MACD, an MACD method with *α* = 0.29 and *β* = 0.04 is also simulated.

Figure 5(c) presents the effect of *α* and *k* on the momentum value. A larger *α* causes momentum to drop quickly as the MN moves away from the AP but causes momentum to slowly rise as the MN approaches the AP. In addition, MRSS can detect user motion faster than MACD when the same value of *α* is applied. Figure 5(d) shows the variation of the momentum value when a user is moving in a Mobile WiMAX environment at a speed of 13 m/s. The simulation results also confirm that the use of a suitable *α* and *k* can improve the quality of user motion detection service.

**Figure 6.**
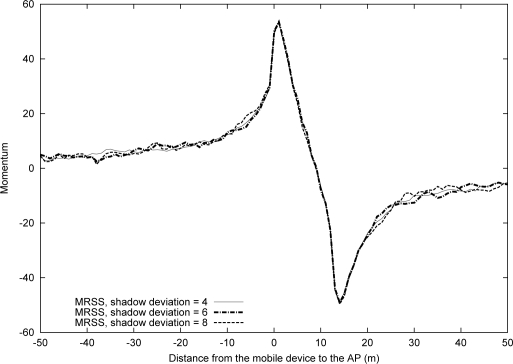
The effect of shadow deviation when *α* = 0.16, *k* = 32 in the WLAN environment.

**Figure 7.**
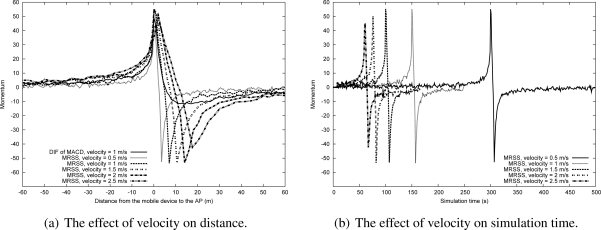
The effect of velocity in the WLAN environment.

Figure 6 shows the effect of shadowing deviation on the momentum value as the MN moves from location A to location B at 1 m/s in a WLAN environment (*α* = 0.16*, k* = 32). The simulation result reveals that MRSS can eliminate all RSS fluctuations. Figures 7(a) and 7(b) show the impact of various velocity on the momentum value for the given movement trajectory when the user is in a WLAN environment. The results indicate that higher velocity corresponds to a greater rate of momentum change.

### 4.2. The Impact of Approaching and Leaving Movement

This subsection analyzes the effect of the start point of an MN on the sensing indicator DIF in MACD [27] and momentum in MRSS when the MN is approaching and leaving its associated AP respectively. In the simulation, an MN moves at 1 m/s in a WLAN environment and at 13 m/s in a WiMAX environment as shown in Figure 4(a). Table 2 shows the configured radio propagation parameters. In order to identify the user motion, the approach threshold is set as 1 dBm and the leaving threshold is set as −1 dBm. When the value of DIF or momentum is larger than the approaching threshold, the user motion is recognized as the approaching state. When the value of DIF or momentum is lower than the leaving threshold, the user motion is recognized as the leaving state. We configure *α*, *β* and *k* as *α* = 0.16 and *β* = 0.04 in MACD and as *α* = 0.16 and *k* = 32 in MRSS.

**Figure 8.**
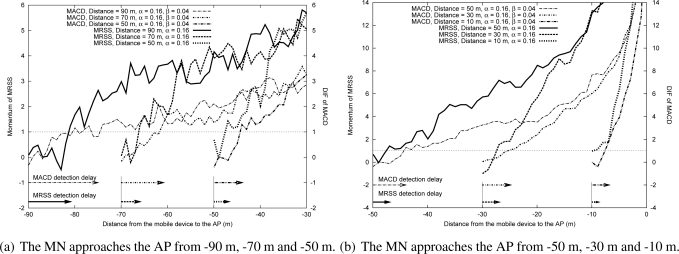
The effect of approaching movement in the WLAN environment.

**Figure 9.**
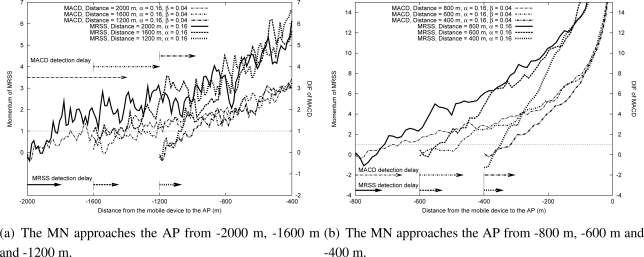
The effect of approaching movement in the Mobile WiMAX environment.

Figures 8(a) and 8(b) show the effect of DIF and momentum when an MN approaches an AP in WLAN. Figures 9(a) and 9(b) show the effect of DIF and momentum when an MN approaches an AP in a Mobile WiMAX environment. The results demonstrate that DIF and momentum are related to the starting position when the MN approaches the AP. When the distance between an MN and its associated AP is large, the value of the measured RSS is small, which results in a small change of the measured RSS. On the other hand, when the MN is close to the AP, the change of the measured RSS is large. As shown in Figures 8(a)–9(b), the momentum value can quickly cross the approaching threshold as the distance between the MN and the AP decreases. That is, the MRSS method can easily detect the user motion. Moreover, the approaching motion detection speed of the MRSS method is faster than that of the MACD method.

**Figure 10.**
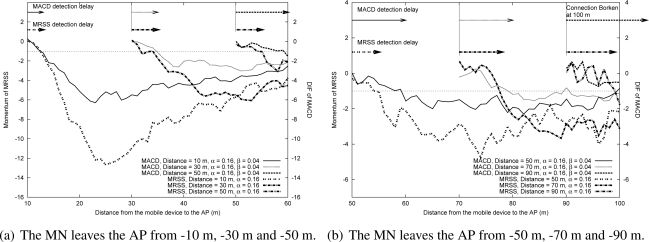
The effect of leaving movement in the WLAN environment.

**Figure 11.**
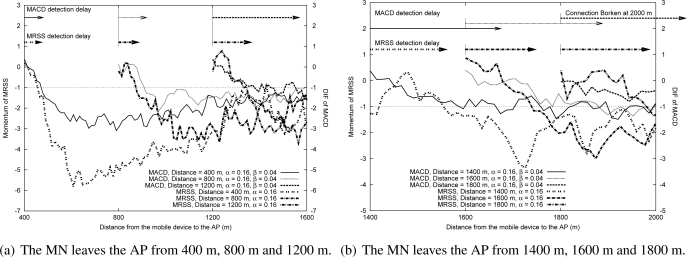
The effect of leaving movement in the Mobile WiMAX environment.

Figures 10(a)–11(b) show the measured DIF and momentum when an MN leaves an AP in a WLAN environment and in a Mobile WiMAX environment respectively. These results also display that the rate of DIF and momentum change declines when the starting position of a user is farther away from the AP, which results in a larger delay for both MACD and MRSS to detect the leaving of the MN. Besides, the simulation results also prove that MRSS can detect the user leaving state faster than MACD. In Figures 10(b) and 11(b), the MACD method can not quickly detect the leaving state of the MN, thus the MN can not trigger a handoff before it moves out the cell.

In the above simulation, the radio propagation strongly affects the behavior of MRSS. As the distance between the MN and the AP increases, the sensitivity of MRSS with a fixed *α* and *k* declines. This property also confirms that the use of a dynamic *α* and *k* for identifying the user motion is necessary.

### 4.3. Feasibility Analysis

**Figure 12.**
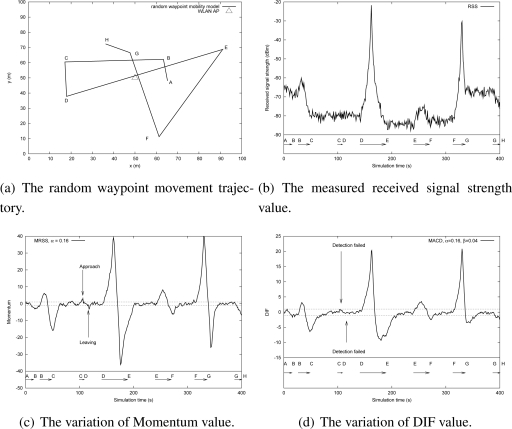
The feasibility analysis of MRSS in the WLAN environment.

**Figure 13.**
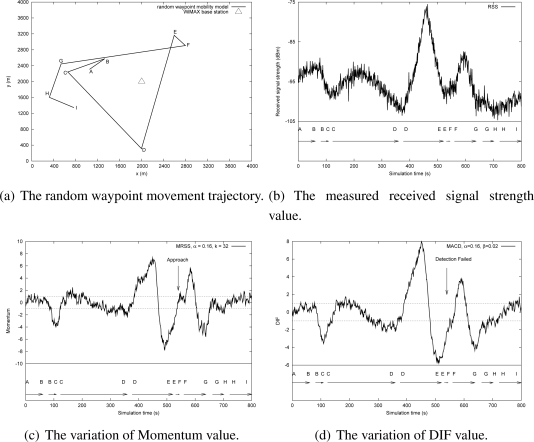
The feasibility analysis of MRSS in the Mobile WiMAX environment.

In this subsection, a random waypoint mobility model was adopted to study the feasibility of MRSS motion detection service in a WLAN and a Mobile WiMAX environment. The WLAN and the Mobile WiMAX environment were configured as shown in Table 2. Figure 12(a) shows the user motion trajectory in a WLAN environment and Figure 13(a) presents the user movement in a Mobile WiMAX environment. The detailed user motion parameters of Figures 12(a) and 13(a) are described in Tables 3 and 4 respectively. According to the analysis in Section 4.2, the distance between an MN and an AP strongly affects the measured RSS. Figure 12(b) plots the measured RSS value from the MN in a WLAN environment. Clearly, when the MN is far away from the AP, the change of RSS is small. For instance, when the MN moves from location C to location D at 2.11 m/s, the change of the measured RSS is not easy to identify.

Figure 12(c) shows the fluctuation of momentum and the results of user motion identification by using the MRSS method. The symbols on the X-axis indicate the locations presented in Figure 12(b). In Figure 12(c), the momentum value can easily determine the user motion state: stationary, leaving or approaching. Figure 12(d) shows the measured DIF value and the motion detection results of the MACD approach. In Figure 12(d), MACD can detect almost all user motion except when the MN moves from location C to location D. It is because the changes of RSS are small and MACD is not sensitive enough to detect them. Figure 13(b) shows the measured RSS of an MN in the Mobile WiMAX environment. The motion detection results of MRSS and MACD are presented in Figures 13(c) and 13(d) respectively. The simulation results also demonstrate that MRSS can detect all user motion correctly and MRSS is more sensitive than MACD.

### 4.4. Ping-Pong Effect Analysis

**Figure 14.**
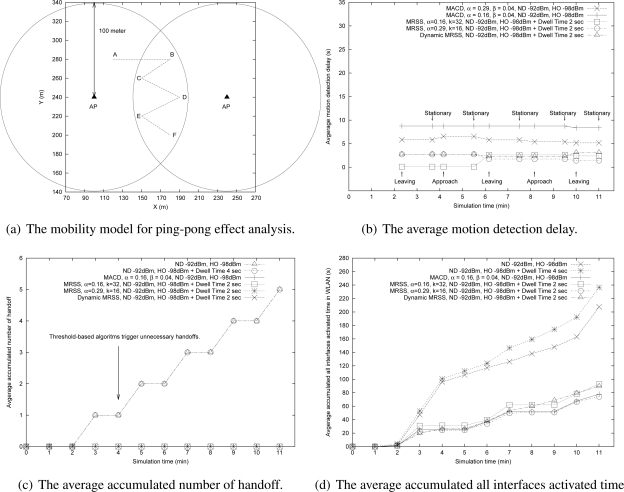
The performance analysis of ping-pong effect.

The frequent handoff of an MN back and forth between two APs is called the ping-pong effect. The ping-pong effect causes many unnecessary handoffs, increases the handoff overhead and reduces the quality of communication services. In this subsection, the impact of the ping-pong effect was evaluated with the trajectory as shown in Figure 14(a). The moving pattern is detailed in Table 5. In this trajectory, the MN does not need to process any handoff procedure. However, when many traditional threshold-based handoff algorithms are used, they may perform various numbers of handoff.

Figures 14(b) and 14(c) illustrate the motion detection delay and motion detection success rate of MRSS, DMRSS and MACD. The results show that the MRSS method and the DMRSS method can accurately detect the user motion and MRSS performs better than the MACD method.

According to the user motion state, the proposed motion detection services can terminate network discovery process to save power and will not trigger any handoff when MN is in a stationary state. Figure 14(c) depicts that the traditional threshold-based handoff algorithms trigger many unnecessary handoffs. Figure 14(d) also indicates that the proposed MRSS and DMRSS motion detection algorithms can reduce unnecessary power consumption.

### 4.5. Evaluation of MRSS-based Handoff Algorithm

In this analysis, the performance of MRSS-based and DMRSS-based handoff algorithms were evaluated by a hexagonal cell based topology as shown in Figure 4(b). In the Mobile WiMAX environment, the size of simulation topology is 9000 m^2^ and the distance between two adjacent APs is 1800 m. A random waypoint mobility model was adopted to simulate a user movement trajectory. Table 2 shows the radio propagation parameters and Table 6 shows the detailed parameters for the mobility model in the simulation.

The performance of MRSS-based handoff method and DMRSS-based handoff method were compared with various RSS threshold-based handoff algorithms [9, 10], the geographic-based handoff algorithm [23] and the MACD-based handoff algorithm [27].

The RSS threshold-based handoff algorithms use two mechanisms, hysteresis (*H*) and dwell-time (*D*), to avoid the ping-pong effect. In the basic RSS threshold-based handoff algorithm (*T*), an MN triggers a handoff when the RSS of the current servicing base station (*RSS_old_*) is lower than a predefined handoff threshold (*T H_HO_*) and the RSS of a neighborhood base stations (*RSS_new_*) is higher than *RSS_old_*. The hysteresis mechanism avoids the ping-pong effect by changing the handoff trigger condition as *RSS_new_ > RSS_old_* + *H*. On the other hand, the dwell time is the time interval that an MN stays in the overlapped area of two APs. The handoff trigger condition must be satisfied and kept over the dwell time, then the MN is allowed to trigger a handoff. The RSS threshold based handoff algorithm can apply the dwell time mechanism and the hysteresis mechanism simultaneous. In the geographic-based handoff algorithm, an MN initiates a handoff according to a GPS and a location information server.

In the simulation, a pre-processing delay was introduced to represent the latency of the network discovery procedure including the time required to turn on the interfaces, scan channels, associate with a chosen AP, etc. The parameters and thresholds of various approaches are presented in Table 7. Each case was simulated 1000 times.

The simulations evaluated the performance of MRSS and DMRSS in terms of motion detection delay, power consumption, total number of handoff and total number of failure handoff.

- **Motion detection delay**: The handoff delay strong impacts the quality of communications. If a motion detection method can detect the motion state quickly, an MN can have sufficient time to process handoff and can stop network discovery immediately when user becomes stationary.
- **Power consumption**: In the network discovery, an MN turns on all its interfaces to search for new base stations and exchange the control information between mobility controllers. This procedure will consume much battery power. A larger accumulated all interfaces activated time represents larger power consumption.
- **Number of handoff**: In some real-time applications, such as voice and video, delay and jitter will damage their quality. The transmission of the applications may be stopped for a while when an MN executes handoff. Unnecessary handoffs may decrease the transmission performance and the quality of the applications.
- **Number of failure handoff**: A handoff may fail if an MN starts the handoff procedure too late. Moreover, fading may cause the variation of RSS and lead to unnecessary handoffs. It may increase the risk of broken connection due to handoff failure and damage the performance of applications.

**Figure 15.**
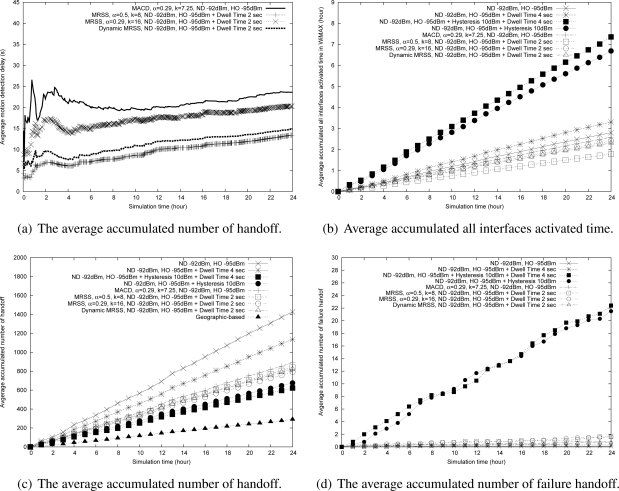
The performance analysis of MRSS-based handoff algorithm in the Mobile WiMAX environment.

The performance evaluation results of various handoff algorithms in the mobile WiMAX environment are shown in Figures 15(a)–15(d). Figure 15(a) illustrates the average motion detection delay of MACD, MRSS and dynamic MRSS. In Figure 15(a), MACD has the largest motion detection delay because it is less sensitive than MRSS. The MRSS with a larger smooth factor (*α*) can detect the user motion faster than the MRSS with a small smooth factor. Using a larger *α* in MRSS can speed the response to the change of RSS, which results in fast motion detection but also unnecessary handoffs. When the RSS of an MN is lower than the predefined network discovery threshold (*T H_ND_*) and the MN moves toward the edge of the cell in a short distance then stops, the MRSS algorithm always triggers network discovery and makes a handoff because it can quickly predict the leaving state. On the other hand, MRSS with a small *α* results in a high motion detection delay. Thus, using a dynamic smooth factor is a better approach.

Figure 15(b) shows the accumulated active time of all interfaces in various approaches. In Figure 15(b), the traditional RSS threshold-based methods consume more power than motion detection-based handoff approaches. In the RSS threshold-based handoff method and the hysteresis combined methods, an MN turns on all interfaces to search for available access networks and executes handoff procedure only according to *T H_ND_*, *T H_HO_* and hysteresis. In addition, the dwell time methods require an MN to remain in the network discovery mode over the dwell time. Thus, these methods consume more power than the motion detection-based approaches. Moreover, the MRSS-based handoff method can identify the user motion state quickly. The MRSS with a larger *α* can detect the user motion state faster than the MRSS with a small *α*, MACD and dynamic MRSS.

Figure 15(c) illustrates the accumulated number of handoff. In Figure 15(c), the geographic-based handoff method has the lowest number of handoff because it triggers the handoff process according to the GPS information. The RSS threshold based handoff algorithm causes the largest number of handoff because the MN always triggers handoffs even when the MN is in a stationary state. The dwell time combined methods and the hysteresis combined methods limit the handoff trigger by a dwell time constraint and a hysteresis constraint, thus the MN triggers handoff late and makes a slightly lower number of handoff than that of the motion detection based handoff algorithm. However, they need to consume more power for network discovery.

MACD, MRSS and DMRSS trigger a handoff based on the user motion state, which result in a lower number of handoffs. A fast motion detection service can quickly trigger a handoff when the MN is leaving and quickly terminate a handoff process when the MN is stationary. Thus, DMRSS and MRSS perform better than MACD.

Figure 15(d) presents the accumulated number of failure handoff. The hysteresis combined methods have a higher number of failed handoff because an MN starts the network discovery process late. The motion detection based handoff approaches have a lower number of failed handoff.

## 5. Conclusions and Future Works

This paper proposed a sensitive motion detection approach called Momentum of Received Signal Strength (MRSS). MRSS can predict an MN’s motion without any assistance from positioning systems. It defines three motion states of an MN: approaching state, stationary state, and leaving state. With MRSS, an MN can trigger a handoff at the right time according to its motion. Thus, MRSS can reduce unnecessary power consumption during handoff procedures. Additionally, a Dynamic MRSS (DMRSS) algorithm is presented to improve the sensitivity of MRSS by using different parameter settings at different motion states. Simulation results show that MRSS and DMRSS perform better than other algorithms. Moreover, the MRSS and DMRSS algorithm are simple and can be easily implemented and integrated in many mobile applications, such as intelligent transport systems (ITS), mobile sensor systems, and vehicular communication systems.

Although DMRSS performs well by using a motion state dependent smooth factor switching method, the suitable smooth factor pairs must be selected manually in advance. In the real world, the network environment (e.g., frequency band, antenna height, transmitter power, antenna gain, fadding model) and the movement of mobile nodes (e.g., velocity, direction) change dynamically. Thus the manual configuration can be selected only for some particular communication systems. In the future, we will continue to develop an intelligent learning algorithm that can self-adjust the weight of various EWMA filters and automatically combine the analysis results from these EWMA filters to identify the user motion state in heterogeneous networks.

## Figures and Tables

**Table 1.** Relationship between the moving average window size *k* and smooth factor *α*.

k	*α*	k	*α*
2	0.95	64	0.08
4	0.80	128	0.05
8	0.50	256	0.04
16	0.29	512	0.02
32	0.16	1024	0.01

**Table 2.** Default parameters for radio propagation.

Wireless Environment	WLAN	Mobile WiMAX [35]
Cell Radius (m)	100	2000
Frequency (Hz)	2.472e9	2.5e9
Path Loss Exponent	4.0	3.5
Shadowing Deviation (dB)	4.0	4.0
Transmitter Antenna Height (m)	1	32
Receiver Antenna Height (m)	1	1.5
Tx Power (dBm)	22	40
Transmitter Antenna Gain (dB)	1	15
Receiver Antenna Gain (dB)	1	1
Rx Sensitivity (dBm)	−101	−101
Sampling Interval (s)	0.05	0.05
Sampling Size	8	10

**Table 3.** The user motion parameters for feasibility analysis in a WLAN environment.

Start	End	Duration (s)	Velocity (m/s)
A	B	14.9336	0.9588
B	B	11.4293	0
B	C	21.8569	2.1112
C	C	50.8848	0
C	D	9.44139	2.3986
D	D	32.2961	0
D	E	47.992	1.6562
E	E	51.0714	0
E	F	29.6391	2.1844
F	F	43.2983	0
F	G	22.9467	2.483
G	G	50.9586	0
G	H	13.2519	0.981

**Table 4.** The user motion parameters for feasibility analysis in a WiMAX environment.

Start	End	Duration (s)	Velocity (m/s)
A	B	60.7216	6.3783
B	B	20.2616	0
B	C	27.4318	27.3668
C	C	14.9154	0
C	D	235.8984	9.955
D	D	17.4647	0
D	E	144.077	20.2115
E	E	10.3044	0
E	F	12.1474	26.9875
F	F	16.7168	0
F	G	77.5418	29.7336
G	G	23.7228	0
G	H	39.9845	21.6865
H	H	21.2732	0
H	I	77.5386	6.7055

**Table 5.** The user motion parameters for the ping-pong effect analysis in WLAN.

Start	End	Duration (s)	Velocity (m/s)
A	B	80	1
B	B	60	0
B	C	80	1.21
C	C	30	0
C	D	80	1.12
D	D	40	0
D	E	80	1.12
E	E	40	0
E	F	30	1.2

**Table 6.** Parameters for the mobility models in the handoff evaluation.

Environment	WiMAX
Mobility model	Random waypoint
Velocity (m/s)	7 – 30
Max Pause (s)	30
Duration (hour)	24
Topology Size	9000 m^2^
Distance between AP	1800 m

**Table 7.** Parameters for the evaluation of handoff mechanisms in WLAN and Mobile WiMAX.

Handoff method	MRSS	MRSS	DMRSS	MACD	T	T + D	T + H + D	T + H	G
*α*	0.29	0.5	0.29/0.5	0.29	—	—	—	—	—
*β*	—	—	—	0.04	—	—	—	—	—
*k*	16	8	16/8	16/256 *	—	—	—	—	—
*T H_P_* (dBm)	1	1	1	1	—	—	—	—	—
*T H_N_* (dBm)	−1	−1	−1	−1	—	—	—	—	—
*T H_ND_* (dBm) in WLAN	−92	−92	−92	−92	−92	−92	−92	−92	—
*T H_HO_* (dBm) in WLAN	−98	−98	−98	−98	−98	−98	−98	−98	—
*T H_ND_* (dBm) in WiMAX	−92	−92	−92	−92	−92	−92	−92	−92	—
*T H_HO_* (dBm) in WiMAX	−95	−95	−95	−95	−95	−95	−95	−95	—
*T H_Dwell_* (s)	2	2	2	—	—	4	4	—	—
Hysteresis (dBm)	—	—	—	—	—	—	10	10	—

T: Threshold / D: Dwell-time / H: Hysteresis / G: Geographic

* In MACD, two EWMA filters (*α* = 0.29*, k* = 16) and (*β* = 0.04*, k* = 256) are used.
